# Difference in topographic morphology of optic nerve head and neuroretinal rim between normal tension glaucoma and central retinal artery occlusion

**DOI:** 10.1038/s41598-022-14943-y

**Published:** 2022-06-28

**Authors:** Ji-Ah Kim, Eun Ji Lee, Tae-Woo Kim, Se Joon Woo

**Affiliations:** 1 Department of Ophthalmology, Ewha Womans University College of Medicine, Ewha Womans University Seoul Hospital, Seoul, Korea; 2 Department of Ophthalmology, Seoul National University Bundang Hospital, Seoul National University College of Medicine, 82, Gumi-ro, 173 Beon-gil, Bundang-gu, Seongnam, 463-707 Gyeonggi-do Korea

**Keywords:** Optic nerve diseases, Retinal diseases

## Abstract

Although central retinal artery occlusion (CRAO) has its own defining pathomechanism and clinical characteristics, morphologic feature of the optic nerve head (ONH) during its later stage is not diagnostic, which makes it difficult to differentiate CRAO from other optic neuropathies. This cross-sectional study was performed to investigate the differences in the topographic morphology of the ONH in eyes with normal-tension glaucoma (NTG) and CRAO. Thirty-one eyes with NTG; 31 eyes with CRAO; and 31 healthy fellow eyes of the subjects with CRAO were included. ONH morphology was evaluated by measuring horizontal rim width (HRW), minimal rim width in the selected horizontal image (MRW), and lamina cribrosa curvature index (LCCI) in horizontal B-scan images obtained using enhanced depth-imaging optical coherence tomography. HRW was smaller and LCCI was larger in NTG eyes than in both CRAO and healthy fellow eyes (both *P* < 0.001), while both were comparable between CRAO and healthy fellow eyes. MRW differed significantly among the three groups, being smallest in NTG eyes followed by CRAO and healthy fellow eyes (*P* < 0.001). NTG and CRAO eyes with a similar degree of RNFL loss differed in ONH morphology, indicating that mechanisms of ONH damage differ between these two conditions.

## Introduction

Central retinal artery occlusion (CRAO) is an ocular emergency that causes acute inner retinal ischemia leading to extensive loss of vision. The incidence of CRAO is estimated around 1.9/100,000 in the United States^1^. CRAO is referred to as a small-vessel stroke, sharing common atherosclerotic risk factors with cardiovascular diseases such as systemic stroke and ischemic heart disease. However, unlike cardiovascular diseases, thrombolytic therapy is known to play only limited role in the treatment of CRAO, and no consensus has been made regarding the optimal treatment of CRAO^2,3^. Characteristics of CRAO revealed by optical coherence tomography (OCT) include inner retinal edema during the acute phase, followed by thinning of the inner retina during subsequent phases^4–7^. Ischemic thinning of the inner retinal layer after resolution of the acute phase has been associated with loss of neural tissue in the optic nerve head (ONH), causing neuroretinal rim (NRR) thinning or pallor, eventually increasing optic disc cupping. Although clinical manifestations are distinct during the acute phase of CRAO, morphologic changes of the ONH during later stages are not indicative of prior changes, making it difficult to differentiate CRAO from other optic neuropathies.

Glaucoma is a progressive optic neuropathy characterized by a typical pattern of optic nerve damage and visual field (VF) loss. It is the leading cause of blindness affecting more than 70 million people worldwide with approximately 10% of them being bilaterally blind^8^. Although the pathogenesis of glaucoma is not fully understood, elevated intraocular pressure (IOP) is considered the most potent factor underlying glaucomatous optic nerve damage^9,10^, and so the treatment strategies concentrate mainly on lowering of IOP. A glaucomatous optic disc is characterized by excavation of the optic cup and thinning of the NRR. The former change is associated with deformation and posterior movement of the lamina cribrosa (LC), which is thought to be induced by IOP related stress^11–13^. The latter change has been reported to result from the progressive loss of prelaminar neural tissues, which is caused by the mechanical stress associated with the structural change of the LC^14–16^. In most cases, glaucomatous cupping is a combination of these two components, reflecting both damage to and remodeling of the laminar connective tissues and progressive loss of retinal ganglion cell axons. Changes occurring during early stages of glaucoma are morphologically distinctive. During much later stages, however, optic nerve damage progresses, resulting in severe excavation of the optic disc and total loss of the NRR, making it difficult to accurately differentiate glaucomatous from nonglaucomatous conditions.

Although structural changes in the ONH have been extensively studied in glaucoma, they have not been well characterized in CRAO. Given the similar morphologic features of the ONH in eyes with advanced stage glaucoma and CRAO, the present study sought to determine the morphologic characteristics of the ONH of each condition and to compare these characteristics in eyes with normal tension glaucoma (NTG) and CRAO.

## Results

This study initially assessed 121 eyes of 121 patients with NTG and 90 with CRAO. Of these, 59 eyes with CRAO were excluded, 31 because they had incomplete type CRAO, five because they had neovascular glaucoma, 11 because they had a tilted or torted disc, and 12 due to poor visualization of the OCT image. In addition, 76 eyes with NTG were excluded, 47 because they had a tilted or torted disc, and 29 due to poor visualization of the OCT image. After matching for age, IOP at the time of the OCT scan, disc area, and global retinal nerve fiber layer (RNFL) thicknesses, 31 eyes with NTG and 31 with CRAO were included in the study, along with the 31 healthy contralateral eyes in the patients with CRAO. There was excellent interobserver agreement in measurements of horizontal rim width (HRW), minimum rim width in the selected horizontal image (MRW), and LC curvature index (LCCI), with intraclass correlation coefficients (ICCs) of 0.996 (95% confidence interval [CI] 0.994–0.998), 0.998 (95% CI 0.997–0.999) and 0.960 (95% CI 0.940–0.974), respectively. The mean follow-up period for OCT scans after CRAO occurrence was 3.0 ± 2.5 years.

**Figure 1:**
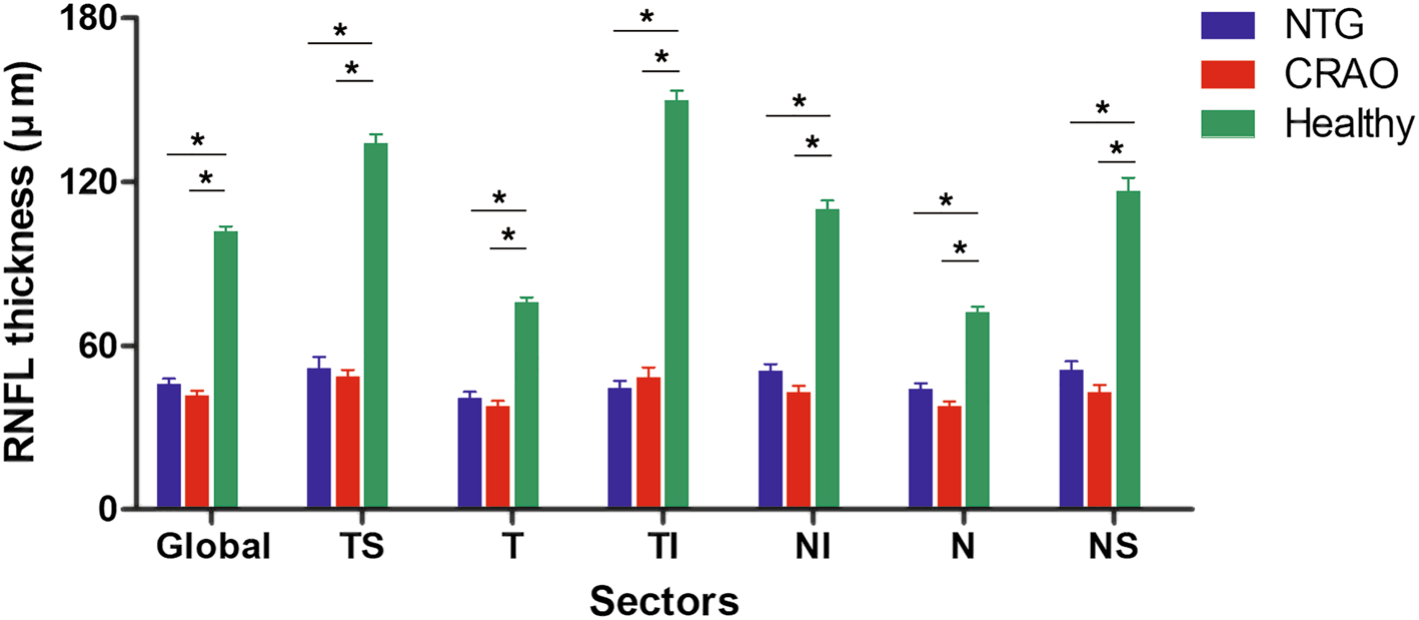
Distribution of circumpapillary retinal nerve fiber layer (RNFL) thicknesses in NTG, CRAO and healthy contralateral eyes. The horizontal bars represent the mean ± standard error of the mean. Asterisks indicate statistically significant differences (*P* < 0.05). RNFL thickness did not differ significantly in any sector of eyes with normal tension glaucoma (NTG) and central retinal artery occlusion (CRAO). Healthy, contralateral eyes of subjects with CRAO; *TS* temporal–superior sector, *T* temporal sector, *TI* temporal–inferior sector, *NI* nasal–inferior sector, *N* nasal sector, *NS* nasal–superior sector.

Table 1 compares the clinical characteristics of the NTG, CRAO, and healthy contralateral eyes of CRAO subjects. Global and sectoral RNFL thicknesses were larger in the healthy contralateral eyes than in both NTG and CRAO eyes (*P* < 0.001 each), but did not differ significantly between the NTG and CRAO eyes (Table 1, Fig. 1).

**Table 1:** Demographic and ocular characteristics of participants (*n* = 93).

Variables	NTG (A) (*n* = 31)	CRAO (B) (*n* = 31)	Healthy (C)* (*n* = 31)	*P*–value	Post–hoc
Age, *years*	62.6 ± 17.0	62.2 ± 17.1	–	0.107	
Male, *n. (%)*	14 (45.2%)	14 (45.2%)	14 (45.2%)	1.000	
Spherical equivalent, *D*	− 0.67 ± 2.25	− 0.05 ± 1.43	− 0.03 ± 1.19	0.822^**†**^	
IOP at OCT, *mmHg*	11.1 ± 2.4	10.9 ± 3.1	10.1 ± 1.9	0.322^**†**^	
VF mean deviation, *dB*	− 21.09 ± 7.03	N/A			
Central corneal thickness, *µm*	549.26 ± 43.86	N/A			
**RNFL thicknesses, *µm***					
Global	46.0 ± 10.4	41.7 ± 9.8	101.6 ± 10.5	** < 0.001**^**‡**^	A = B < C
Temporal–superior	51.5 ± 23.2	48.5 ± 14.3	134.2 ± 18.2	** < 0.001**^**†**^	A = B < C
Temporal	40.9 ± 12.6	37.7 ± 11.0	75.7 ± 11.0	** < 0.001**^**†**^	A = B < C
Temporal–inferior	44.5 ± 14.3	48.4 ± 20.0	149.8 ± 19.7	** < 0.001**^**†**^	A = B < C
Nasal–inferior	50.8 ± 12.9	42.9 ± 13.4	109.9 ± 18.6	** < 0.001**^**‡**^	A = B < C
Nasal	44.2 ± 11.4	37.7 ± 9.7	72.2 ± 11.3	** < 0.001**^**‡**^	A = B < C
Nasal–superior	51.1 ± 17.8	42.9 ± 14.7	116.7 ± 26.7	** < 0.001**^**†**^	A = B < C

Values are shown in mean ± standard deviation or n (%) values, with statistically significant *P*–values in boldface.

*NTG* normal tension glaucoma, *CRAO* central retinal artery occlusion, *IOP* intraocular pressure, *OCT* optical coherence tomography, *VF* visual field, *N/A* not available, *RNFL* retinal nerve fiber layer.

*Healthy fellow eyes of CRAO subjects.

Comparison was performed using Kruskal–Wallis^**†**^ and ANOVA^**‡**^.

**Figure 2:**
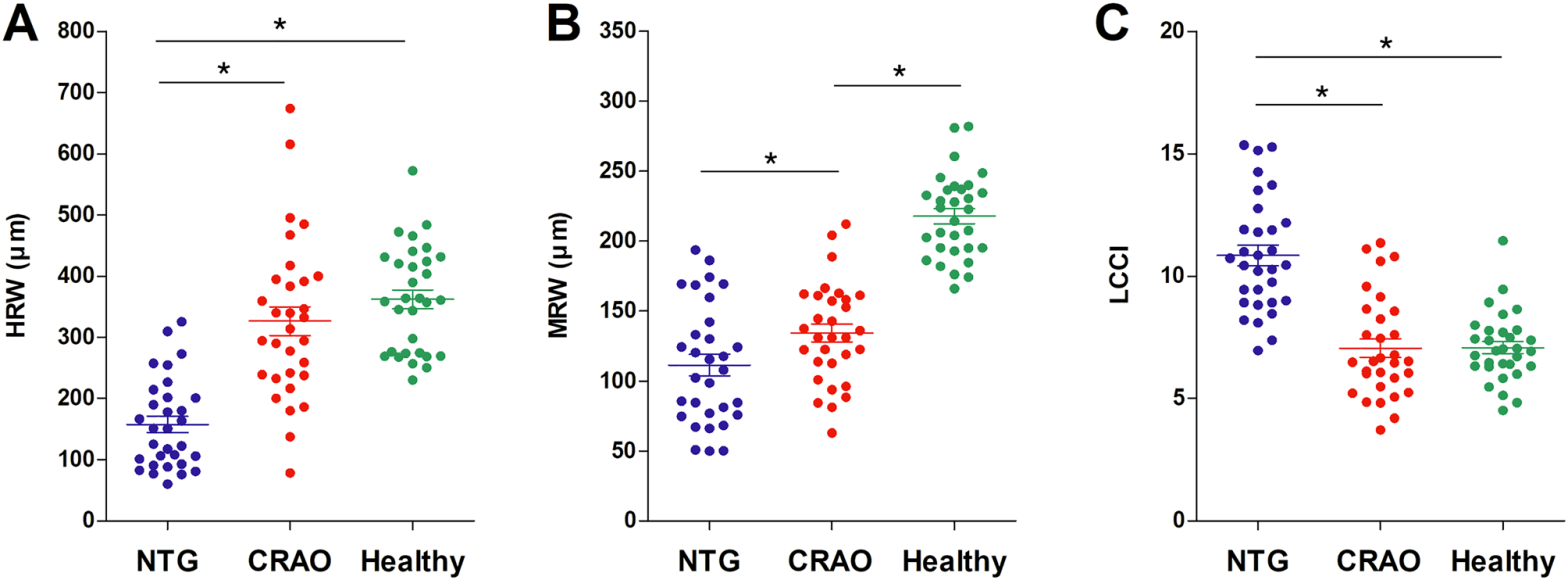
Scatterplots showing horizontal rim width (HRW) (**A**), minimal rim widths (MRW) (**B**), and lamina cribrosa curvature index (LCCI) (**C**) in eyes with normal tension glaucoma (NTG), central retinal artery occlusion (CRAO), and healthy contralateral eyes of subjects with CRAO. HRW and LCCI differed significantly in CRAO and NTG eyes, but did not differ in CRAO and healthy contralateral eyes (**A**,**C**). MRW differed significantly in the three groups (**B**). The horizontal bars represent the mean ± standard error of the mean. Asterisks indicate statistically significant differences (*P* < 0.05).

**Figure 3:**
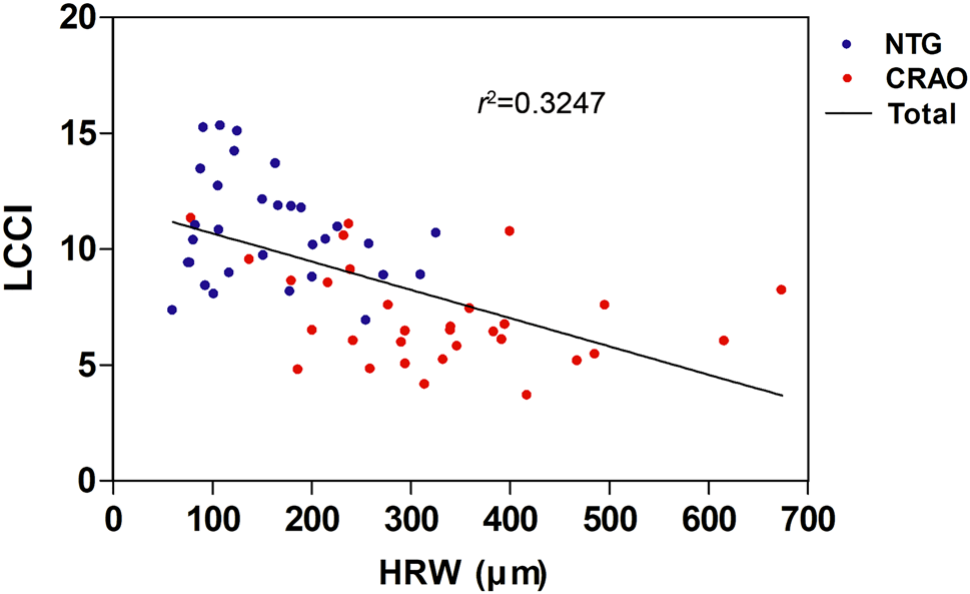
Scatter plot showing the relationship between the horizontal rim width (HRW) and the lamina cribrosa curvature index (LCCI) in normal tension glaucoma (NTG) and central retinal artery occlusion (CRAO) eyes. Overall, a larger HRW was associated with a smaller LCCI (*P* < 0.001, *r*^2^ = 0.3247) (solid line).

Table 2 and Fig. 2 show comparisons of ONH morphology in the three groups of eyes. Disc area and disc ovality did not differ significantly among these groups (*P* ≥ 0.137). HRW, however, was significantly smaller in NTG eyes than in both CRAO and healthy contralateral eyes (*P* < 0.001), but did not differ significantly between CRAO and healthy contralateral eyes. MRW differed significantly among all three groups, being smallest in NTG eyes, followed by CRAO eyes and healthy contralateral eyes (*P* < 0.001). Horizontal-to-minimum rim width ratio in the selected horizontal image (HMR) was smallest in NTG eyes followed by healthy contralateral eyes and CRAO eyes (*P* < 0.001). LCCI was significantly larger in NTG eyes than both CRAO and healthy contralateral eyes (*P* < 0.001), but did not differ significantly in the latter two groups. Overall, smaller HRW was associated with larger LCCI (*P* < 0.001, *r*^2^ = 0.3274; Fig. 3) in NTG and CRAO eyes. However, subanalyses within each group found that these associations were not significant.

**Table 2:** Comparison of optic nerve head morphology between the groups (*n* = 93).

Variables	NTG (A) (*n* = 31)	CRAO (B) (*n* = 31)	Healthy (C)* (*n* = 31)	*P*–value	Post–hoc					
					A–B		B–C		C–A	
Disc area, *mm*^*2*^	2.56 ± 0.41	2.55 ± 0.46	2.50 ± 0.45	0.915^**†**^						
Disc ovality	1.05 ± 0.07	1.09 ± 0.10	1.05 ± 0.08	0.137^**‡**^						
Mean HRW, *µm*	157.6 ± 73.1	326.6 ± 130.8	362.2 ± 85.8	** < 0.001**^**†**^	** < 0.001**		0.153		** < 0.001**	A < B = C
Mean MRW, *µm*	111.4 ± 42.8	134.3 ± 35.6	217.9 ± 29.9	** < 0.001**^**‡**^	**0.041**		** < 0.001**		** < 0.001**	A < B < C
Mean HMR	1.3 ± 0.2	2.3 ± 0.7	1.6 ± 0.3	** < 0.001**^**†**^	** < 0.001**		** < 0.001**		** < 0.001**	A < C < B
Mean LCCI	10.8 ± 2.3	7.1 ± 2.1	7.1 ± 1.4	** < 0.001**^**‡**^	** < 0.001**		1.000		** < 0.001**	B = C < A

Values are shown in mean ± standard deviation, with statistically significant *P*–values in boldface.

*NTG* normal tension glaucoma, *CRAO* central retinal artery occlusion, *HRW* horizontal rim width, *MRW* minimum rim width, *HMR* horizontal-to-minimum rim width ratio, *LCCI* lamina cribrosa curvature index.

*Healthy fellow eyes of CRAO subjects.

Comparison was performed using Kruskal–Wallis^**†**^ and ANOVA^**‡**^.

To eliminate the influence of large retinal vessels, which are more prominent at the nasal rim, measurements obtained in the temporal area were compared in the three groups (Table 3). The results did not differ substantially from those shown in Table 2.

**Table 3:** Comparison of neuroretinal rim widths in the temporal area between the groups (*n* = 93).

Variables	NTG (A) (*n* = 31)	CRAO (B) (*n* = 31)	Healthy (C)* (*n* = 31)	*P*–value	Post–hoc				
					A–B		B–C	C–A	
Mean temporal HRW, *µm*	147.1 ± 80.7	298.3 ± 135.6	342.1 ± 98.0	** < 0.001**^**†**^	** < 0.001**		0.248	** < 0.001**	A < B = C
Mean temporal MRW, *µm*	98.9 ± 43.0	126.8 ± 37.0	191.5 ± 28.9	** < 0.001**^**†**^	**0.010**		** < 0.001**	** < 0.001**	A < B < C
Mean temporal HMR	1.5 ± 0.3	2.3 ± 0.9	1.8 ± 0.5	** < 0.001**^**‡**^	** < 0.001**		**0.005**	**0.005**	A < C < B

Values are shown in mean ± standard deviation, with statistically significant *P*–values in boldface.

*NTG* normal tension glaucoma, *CRAO* central retinal artery occlusion, *HRW* horizontal rim width, *MRW* minimum rim width, *HMR* horizontal-to-minimum rim width ratio.

*Healthy fellow eyes of CRAO subjects.

Comparison was performed using ANOVA^**†**^ and Kruskal–Wallis^**‡**^.

**Figure 4:**
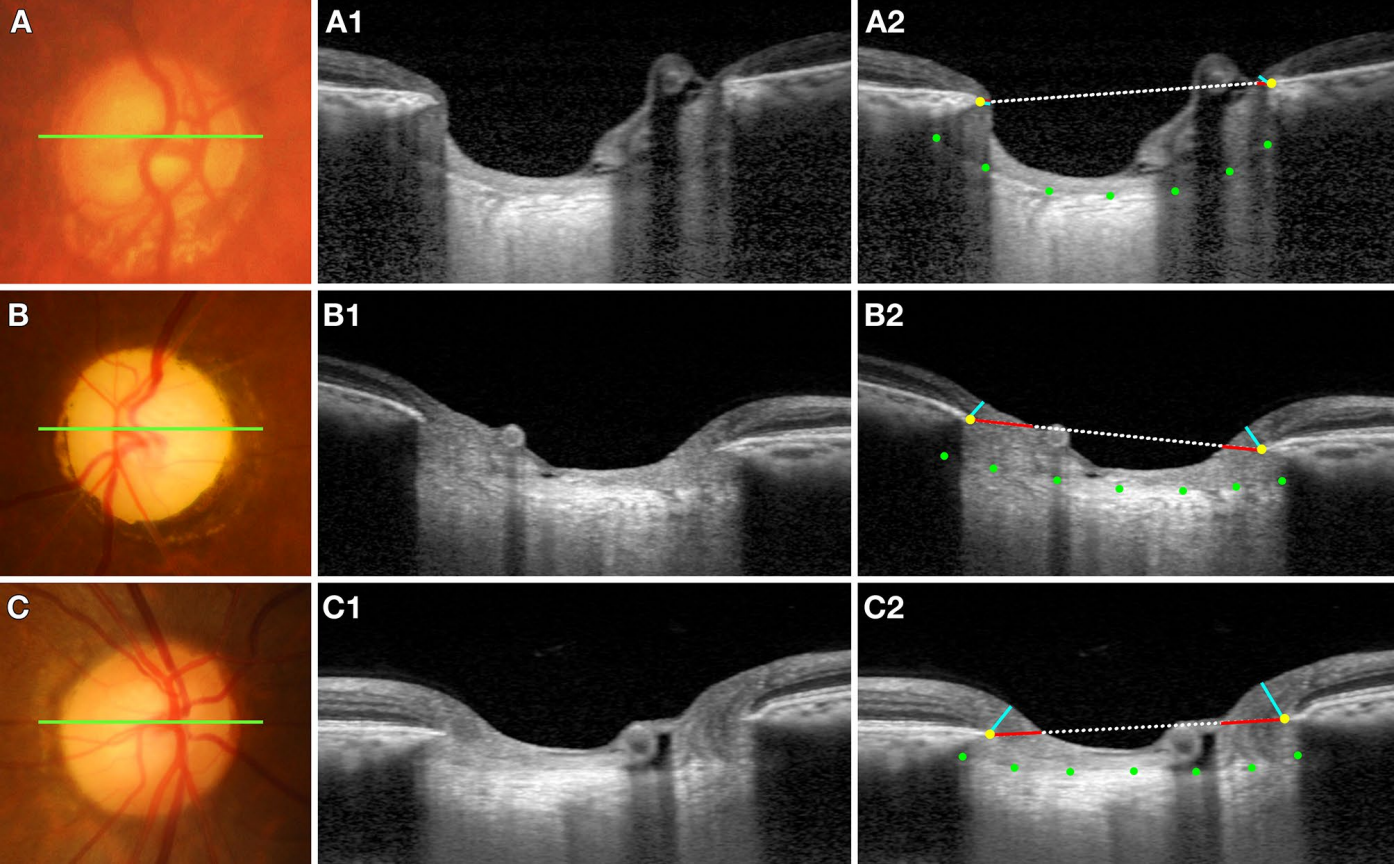
Representative eyes with (**A**) normal tension glaucoma (NTG) and (**B**) central retinal artery occlusion (CRAO), and (**C**) the healthy contralateral eye of the CRAO subject in (**B**). Color disc images (**A**–**C**), B-scan images in the plane indicated by the green lines in the color disc images (**A1**–**C1**). (**A2–C2**) are the same images as (**A1**–**C1**) indicating the horizontal rim width (HRW), minimum rim width (MRW), and lamina cribrosa curve. The HRW (red line) was smaller in the NTG eye than in the CRAO and healthy contralateral eyes; MRW (turquoise line) was largest in the healthy contralateral eye followed by the CRAO and NTG eyes; and the degree of posterior bowing of the anterior lamina cribrosa surface (green dots) was greatest in the NTG eye.

Figure 4 shows eyes with NTG and CRAO matched by age, IOP, optic disc area, and global RNFL thickness, as well as the healthy contralateral eye of the subject with CRAO. HRW and MRW were noticeably small and LCCI noticeably large in the NTG eye. MRW was smaller in the CRAO eye than in its contralateral eye, whereas both HRW and LCCI were comparable in these two eyes.

## Discussion

The present study analyzed features of the ONH in eyes with NTG and CRAO. Small HRW and MRW and large LCCI were characteristic of NTG eyes, whereas CRAO eyes had larger HRW and MRW and smaller LCCI than NTG eyes with similar amounts of RNFL loss. Comparisons of CRAO eyes and healthy contralateral eyes in the same subjects showed that MRW was decreased in CRAO eyes, whereas HRW and LCCI were comparable. Although these findings may be insufficient to differentiate between the two diseases, they may help to understand differences in the pathophysiology of NTG and CRAO. To our knowledge, this is the first study to compare ONH morphology in eyes with NTG and CRAO.

Despite their similar degree of RNFL loss, NTG and CRAO eyes showed significant differences in rim thickness, as determined by both HRW and MRW. Moreover, HRW did not differ between CRAO eyes and healthy contralateral eyes of the same subjects. This finding is consistent with results in rhesus monkey eyes, showing that optic disc cupping was similar in CRAO and normal eyes^17^. When the primary insult is outside the ONH, the ONH glioarchitecture is not disrupted. In response to axonal loss, hypertrophic astrocyte processes fill the space formerly occupied by axons, maintaining the general tissue architecture in non-glaucomatous descending/ascending optic neuropathies^18,19^. In glaucoma, however, disruption and loss of the glioarchitecture through progressive disorganization can cause rim tissue atrophy^20^. Even eyes at early stages of glaucoma showed slight disorganization of the previously ordered arrangement of glial columns^21,22^. Evaluation of experimental glaucoma in primate eyes with rim thinning has shown the destruction of glial columns, marked disarrangement of glial cells, and loss of prelaminar tissues^23,24^.

Although HRW was comparable, MRW was significantly smaller in CRAO eyes than in healthy contralateral eyes. This may be attributable to the site of the MRW measurement, which was anatomically close to the transition zone between the superficial nerve fiber layer (SNFL) and the prelaminar part that includes components from both areas^20^. The NRR has four zones in the anterior ONH: the SNFL, the transitional zone, the anterior prelaminar area, and the posterior prelaminar area^25,26^. The NRR contains two distinct spatial arrangements of astrocyte processes, one parallel and the other perpendicular, extending towards the course of axon bundles. In the RNFL–SNFL compartment containing parallel axons and glial processes there is no spacer between the upper and lower layers of axon bundles^27–29^, resulting in a decrease in rim width that is relatively proportional to the decrease in axons. The compact arrangement of bundles becomes less parallel and disperses when these bundles reach the optic disc, bending at the transition zone^30^. Therefore, the tissue thickness in this area is less affected by the decrease in axons.

The combination of a relatively small MRW and a relatively large HRW in CRAO eyes made their ratio (HMR) the most distinguishable parameter characterizing ONH morphology in CRAO. A large HMR indicates that the NRR was relatively well maintained despite the amount of axonal loss. Additional studies are required to determine whether a large HMR could serve as a specific indicator of ONH morphology in eyes with CRAO.

Morphologically, the ONH in eyes with NTG was characterized by a large LCCI. Glaucoma is caused by mechanical stresses on the ONH, with the LC regarded as the principal site of axonal injury of retinal ganglion cells^31,32^. Deformation of the LC is thought to induce damage in retinal ganglion cells by blocking axonal transport, thereby reducing the diffusion of nutrients from the laminar capillaries to the adjacent axons^33,34^, or by connective tissue remodeling^35^. LC deformation has been regarded as an important pathophysiologic manifestation of glaucoma, even when accompanied by low IOP^36,37^. The difference between LC curves of NTG and CRAO eyes with similar IOP provides additional evidence for the difference in their pathomechanisms. These findings also suggest that acute retinal ischemia does not induce morphologic changes in the LC.

Global and sectoral RNFL thicknesses were larger in the healthy contralateral eyes of subjects with CRAO than in both CRAO and NTG eyes, but did not differ significantly between the latter two groups. Reductions in inferotemporal and superotemporal RNFL thicknesses are diagnostic feature of glaucoma^38,39^. Although glaucomatous NRR loss can occur in a diffuse manner, it can also occur sequentially in sectors of the eye. Generally, NRR loss was found to begin in the inferotemporal disc region and to progress sequentially to the superotemporal, temporal horizontal, inferior nasal, and superior nasal sectors. Although this sequential progression may be applicable to early glaucoma^40^, the present study included patients with advanced glaucoma, as one of the factors used to match patients with NTG and CRAO was the amount of RNFL loss. The inclusion of advanced glaucoma patients likely masked the sectoral RNFL thickness distribution characteristic of glaucoma.

Overall, HRW was negatively associated with LCCI, suggesting that HRW decreases as the rim tissue turns off along the deformed LC. However, subanalyses within each group found that the associations between HRW and LCCI were not significant. Most NTG eyes had smaller HRW and larger LCCI, whereas CRAO and healthy eyes differed in HRW while having small LCCI. The relatively small sample size of this study could also have contributed to these inconclusive results. Future studies in larger numbers of subjects are needed to evaluate the relationship between HRW and LCCI.

This study had several limitations. First, the sample size of each group was relatively small, primarily because the incidence of CRAO is lower than that of NTG. Second, eyes with tilted or torted optic discs were excluded, making the findings of this study inapplicable to eyes with these conditions. Third, HRW and MRW were measured manually using the horizontal disc scans, primarily because many of the patients included in this study were enrolled before the Bruch’s membrane opening (BMO)-MRW protocol of Spectralis, which is based on the radial disc scans, became available. Horizontal disc scans were more useful than radial scans for evaluation of the LC curve, because the LC has a relatively regular configuration in the horizontal plane, having a flat or U-shaped appearance despite differences in regional steepness, allowing the measurement of LCCI^41,42^. However, using the horizontal images also means that our MRW measurement may not accurately represent the “minimum” rim width. Therefore, it should be referred to as minimum rim width in a selected horizontal image. Fourth, this study included eyes with NTG, preventing the generalization of these results to all eyes with glaucoma. Because IOP has been associated with LC morphology, the bias resulting from the influence of IOP could be ruled out by excluding eyes with high IOP. Future studies should therefore include eyes with glaucoma other than NTG.

In conclusion, the present study found that HRW was larger and LCCI was smaller in both CRAO and healthy contralateral eyes than in NTG eyes, but that these parameters did not differ significantly between CRAO and healthy contralateral eyes. The differences observed in ONH indices likely reflect differences in ONH morphology and in the pathogenesis of these two diseases.

## Methods

### Study subjects

This cross-sectional study included patients with NTG who were enrolled in the Investigating Glaucoma Progression Study (IGPS). IGPS is an ongoing prospective clinical study being conducted by the Seoul National University Bundang Hospital Glaucoma Clinic. This study also included patients with CRAO who visited Retina Clinic of Seoul National University Bundang Hospital from January 2006 to March 2021. The protocol of the present study was approved by the Seoul National University Bundang Hospital Institutional Review Board and conformed to the tenets of the Declaration of Helsinki. Written informed consent was obtained from the patients with NTG. The IRB waived the informed consent for the retrospective review of the charts of patients with CRAO.

Each patient underwent comprehensive ophthalmic examinations, including measurements of best-corrected visual acuity (VA), refraction tests, slit-lamp biomicroscopy, Goldmann applanation tonometry, gonioscopy, and fundus photography (Kowa VX-10, Kowa Medicals, Torrance, CA, USA). Other ophthalmic examinations included scanning of the circumpapillary RNFL and macular and ONH areas using spectral-domain (SD) OCT (Spectralis, Heidelberg Engineering, Heidelberg, Germany), and measurements of corneal curvature (KR-1800, Topcon, Tokyo, Japan). Optic disc area was measured using the built-in manual caliper tool designed to calculate the area on fundus images in Heidelberg Eye Explorer (software version 1.10.4.0, Heidelberg Engineering), a viewer program provided with the Spectralis OCT device. Patients with NTG also underwent standard automated perimetry (Humphrey Field Analyzer II 750, 24–2 Swedish interactive threshold algorithm, Carl Zeiss Meditec, Dublin, CA, USA), and patients with CRAO underwent fluorescein angiography (FA; Kowa VX-10, Kowa Medicals).

NTG was defined as the presence of glaucomatous optic nerve damage (i.e., NRR thinning/notching, and an RNFL defect in the corresponding region), a corresponding glaucomatous VF defect, an open iridocorneal angle on gonioscopic examination, a maximum IOP ≤ 21 mmHg without glaucoma medications, no prior history of long-term use of steroid medications and no identifiable secondary cause of glaucoma. A glaucomatous VF change was defined as the fulfillment of two or more of the following criteria: (1) outside normal limits on the glaucoma hemifield test; (2) three abnormal points with a < 5% probability of being normal, including one abnormal point with a < 1% probability of being normal by pattern deviation, or (3) a pattern standard deviation < 5%. These VF defects were confirmed on two consecutive reliable tests, defined as tests with a fixation loss rate ≤ 20% and false-positive and false-negative error rates ≤ 25% each.

CRAO was diagnosed as the occurrence of classic clinical findings of sudden, painless vision loss, and funduscopic findings indicative of retinal hypoperfusion, as confirmed by FA^43^. CRAO was categorized as incomplete, subtotal, or total^44^. Incomplete CRAO is characterized by diminished VA, slight retinal edema with indefinite cherry-red spots, and mildly delayed retinal arterial perfusion on FA. Subtotal CRAO is identified as severe reduction in VA, distinct retinal edema with cherry-red spots, and severely delayed retinal arterial perfusion. Total CRAO is characterized by severe retinal ischemia and massive retinal edema in the macula, often accompanied by choroidal perfusion delay and no light perception^44^. The present study included patients with subtotal or total CRAO involving one eye, and excluded those with branch retinal artery occlusion. Eyes with maximum IOP > 21 mmHg, family history of glaucoma, or history of using glaucoma medication were also excluded to eliminate any effect of IOP on ONH morphology. Patients with CRAO and NTG were matched 1:1 by age, IOP at the time of OCT, disc area, and global RNFL thickness. The control group consisted of unaffected eyes in subjects with CRAO.

Patients were also excluded if they had a spherical equivalent of <  − 8.0 D or >  + 3.0 D, a cylinder correction of <  − 3.0 D or >  + 3.0 D, a history of intraocular surgery except for uneventful cataract surgery, or any retinal disease, such as diabetic retinopathy or retinoschisis or neurological disease such as a pituitary tumor. Eyes were also excluded if they had optic disc tilt, defined as a tilt ratio (ovality index) of the longest to the shortest diameter > 1.3^45,46^; or torsion, defined as a torsion angle, or deviation of the long axis of the optic disc from the vertical meridian, of > 15°^46,47^. Eyes were excluded when good-quality images (i.e., quality score > 15) could not be obtained for more than five sections of enhanced depth imaging (EDI) SD-OCT disc scans. When the quality score does not reach 15, image acquisition by the Spectralis OCT system stops automatically, and the image of the corresponding section is not obtained.

### Enhanced depth imaging OCT of the optic nerve head

The optic nerve was imaged using the EDI technique of the Spectralis OCT system, a technique originally developed to visualize the full thickness of the choroid^48^. EDI has been shown to yield images with a stronger signal and better image contrast in the deep ONH tissue than conventional imaging techniques^49^. Patients with NTG underwent OCT scans of the ONH prior to the initiation of ocular hypotensive treatment to eliminate any potential effects of IOP on LC morphology^11,50,51^. Patients with CRAO underwent OCT scans at least 6 months after the onset of CRAO, after reduction and stabilization of the acute swelling of the ONH.

Patients were imaged through undilated pupils using a rectangle subtending 10 degrees × 15 degrees of the optic disc. This rectangle was scanned with approximately 75 B-scan section images that were separated by 30–34 µm (the scan line distance was determined automatically by the machine). Approximately 42 SD-OCT frames were averaged for each section. This protocol provided the best trade-off between image quality and patient cooperation^49^. Potential magnification errors were avoided by entering the corneal curvature of each eye into the Spectralis OCT system before scanning.

ONH parameters, including LCCI and NRR width on horizontal B-scan images, were measured by two experienced observers (J.A.K. and E.J.L.), who were masked to subjects’ clinical information. Two observers used the same set of images for the measurement. The mean of the measurements made by the two observers was used for analysis.

### Measurement of neuroretinal rim widths

**Figure 5:**
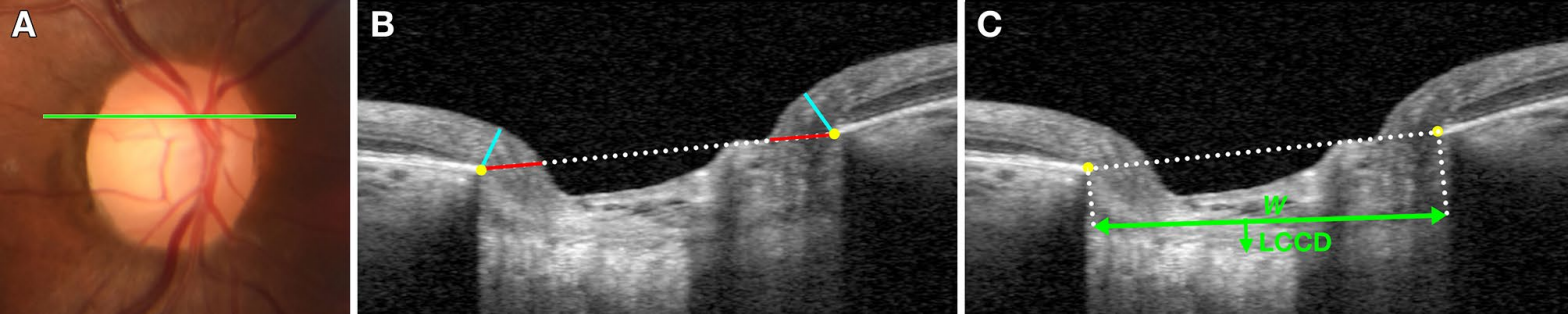
Measurement of optic nerve head indices. (**A**) Color disc photograph; the horizontal green line indicates the plane in which the measurements were made. (**B**) B-scan image obtained at the location indicated by the green line in (**A**). Horizontal rim width (HRW; red line) and minimal rim width (MRW; turquoise line) were measured at the temporal and nasal rims. The HRW was defined as the distance between the projection of Bruch’s membrane opening (BMO, yellow glyphs) to the BMO reference plane and the internal limiting membrane (ILM) along the BMO reference plane. The MRW was defined as the minimum distance between the BMO and the ILM. (**C**) Same image as in (**B**). The lamina cribrosa curvature index was measured by dividing the lamina cribrosa curve depth (LCCD; arrow) within the BMO (yellow glyphs) by the width (W; double-headed arrow) of this opening and then multiplying by 100.

HRW and MRW were measured based on BMO, as had been detailed by Reis et al. (Fig. 5)^52^. HRW was defined as the distance between the projection of BMO to the BMO reference plane and the internal limiting membrane (ILM), along the BMO reference plane, whereas MRW was defined as the minimum distance between the BMO and the ILM. HMR was determined by dividing HRW by MRW (HRW/MRW). If overlying large vessels prevented interpretation of the structures, an adjacent image was used; thus, the thicknesses of these vessels were not included in the measurements. HRW and MRW were measured at three locations (central, and superior and inferior mid-periphery) equidistant across the vertical optic disc diameter using the built-in manual caliper tool in Heidelberg Eye Explorer. The average of three scan values separated by two scan intervals was calculated for each location.

### Measurement of lamina cribrosa curvature index

To quantify the posterior LC curve on the SD-OCT B-scan images, we defined the LCCI as the inflection of a curve representing a section of the LC. LCCI has been recognized as a robust parameter representing the glaucomatous LC deformation^41,53–55^ and was shown to predict progressive RNFL thinning^42,56^.

The method used to calculate LCCI has been described previously^42,53^. In brief, the width (*W*) of the BMO was measured on each B-scan, followed by measurement of the LC curve depth (LCCD). The BMO width was defined as the width of the line connecting the temporal and nasal termination points. Lines were drawn from each BM termination point perpendicular to the BMO reference line, until they met the anterior LC surface. The line connecting the two points on the anterior LC surface was the reference line for measuring the LCCD. The LCCD was determined as the maximum depth from this reference line to the anterior surface (Fig. 5). The LCCI was then calculated as (LCCD/*W*) × 100. Since the curvature was thereby normalized according to LC width, LCCI represents the posterior curvature of the anterior LC surface independent of the actual size of the ONH. Only the LC within the BMO was considered because the LC was often not clearly visible outside of the BMO. LCCI was measured at the three locations on the same B-scans used to measure HRW and MRW, with the built-in manual caliper tool in Heidelberg Eye Explorer.

### Measurement of RNFL thickness

The scan circle around the optic nerve for circumpapillary RNFL scan was 12 degrees in diameter. The accuracy of the segmentation of the RNFL was reviewed and segmentation errors were corrected manually. The Spectralis OCT system divides the circumpapillary scanning circle into six sectors: temporal–superior (46°–90°), temporal (316°–45°), temporal–inferior (271°–315°), nasal–inferior (226°–270°), nasal (136°–225°), and nasal–superior (91°–135°). The global RNFL thickness and the RNFL thickness in each of these sectors were recorded for further analysis.

### Data analysis

Except where stated otherwise, data are presented as mean ± standard deviation. The interobserver agreements for measuring the HRW, MRW, and LCCI were assessed by calculation of ICCs and 95% confidence intervals (CIs). Comparisons between three groups were performed using ANOVA or Kruskal–Wallis test depending on the assumption of normality using Shapiro–Wilk test. Post-hoc analysis of ANOVA was performed using the Tukey test. Comparisons between two groups were analyzed by *t*-tests, Mann–Whitney U-tests, paired t-tests, or Wilcoxon’s signed-rank tests, as appropriate. All statistical analyses were performed using the Statistical Package for the Social Sciences (version 22.0, SPSS, Chicago, IL, USA), with *P* values < 0.05 considered statistically significant. Statistical power of the sample size was calculated by G ∗ power 3.1 and showed 1.31/46.9/0.94 effect size, 0.05/0.05/0.05 α err prob and 1.0/1.0/1.0 power (1 − β err prob) for MRW/HRW/LCCI, respectively^57^.

## Data Availability

The datasets generated during and/or analysed during the current study are available from the corresponding author on reasonable request.
